# *Staphylococcus aureus* Typing by Digestion of Protein A Coding Gene Using Bsp143I

**DOI:** 10.5812/jjm.10320

**Published:** 2014-06-01

**Authors:** Fatemeh Shakeri, Ezzat Allah Ghaemi, Maya Babai Kochkaksaraei

**Affiliations:** 1 Department of Biology, Payame Noor University, Gorgan, IR Iran; 2 Department of Microbiology, Golestan University of Medical Sciences, Gorgan, IR Iran; 3 Danesh Clinical Laboratory, Gorgan, IR Iran

**Keywords:** Staphylococcal Protein A, *Staphylococcus aureus*, Methicillin-Resistant *Staphylococcus aureus*

## Abstract

**Background:**

Protein A is the virulence factors of *Staphylococcus aureus* rolling in its pathogenesis, and its gene is used for typing. Polymerase chain reaction-restriction fragment length polymorphism (PCR-RFLP) with different enzymes has been used for this action.

**Objectives:**

In this study, we used Bsp143I enzyme for digestion of the gene, coding protein A (*spa* gene) in *S. aureus.* The bacteria were isolated from patients and healthy carriers in Gorgan, north of Iran.

**Patients and Methods:**

DNAs of 128 *S. aureus* subjects (53 from healthy carriers and 75 from patients) were extracted and amplified using specific primers of the* spa *gene. The product was digested by Bsp143I enzyme and its pattern was assessed by gel electrophoresis.

**Results:**

There were seven* spa *types among the tested *S. aureus* samples, among which six types differed in the repeated X region of the* spa *gene, but the seventh type had a deletion on one of BSP143I restriction sites. The frequency of* spa *types among isolated *S. aureus* samples as well as healthy carriers was six and five, respectively. *S. aureus* isolated from wounds showed the most diverse* spa *types (five) among clinical samples. Types 1, 2 and 4 were observed in all clinical samples, while only one case of type 3 was identified among patients, whereas this type constituted over 32% of the isolates among carriers. We found seven and four* spa *types among methicillin-resistant *S. aureus* (MRSA) and methicillin-sensitive *S. aureus* (MSSA) isolates, respectively.

**Conclusions:**

Our results showed that typing the* spa *gene using PCR-RFLP using Bsp143I was an acceptable method for typing *S. aureus.* Furthermore, this survey showed that the types in healthy carriers and MSSA were more variable than patient and MRSA isolates, respectively. We used the Bsp143I enzyme, which was not used in any previous studies on the* spa *gene. The results of this study suggested that we can use PCR-RFLP of* spa *gene by Bsp143I for molecular typing and sequencing of *S. aureus*, instead of relatively expensive methods. This method is relatively rapid and inexpensive, and can be accomplished in centers with conventional molecular facilities.

## 1. Background

*Staphylococcus aureus* has remained a primary pathogen of nosocomial and community-acquired infections worldwide. Infection of this bacterium causes disease in different body organs and easily spreads in the hospital. The emergence and spread of methicillin-resistant and Vancomycine- intermediate *S.aureus *(VISA) isolates have increased their importance. Understanding the epidemiology and distribution of this bacterium in the community as well as hospitals is necessary to determine its source, to restrict its spread especially in hospital settings. Finding a suitable marker for this purpose has been considered for many years and different methods have been used. Phage typing is one of the oldest methods, which has been declined in the recent years because of its technical problems and the nature of its usage, and instead, molecular methods on the basis of coagulase gene typing (coa typing),* agr *gene and others have been included. Polymorphism of the gene encoding protein A (*spa*) is one of the methods used in several studies (1).

Protein A is one of the main cell wall proteins considered as the virulence factor of *S. aureus* (2). This protein is unique in this bacterium, as it can act as IgG binding site, except for IgG3. Although each protein A contains five IgG binding sites (A, B, C, D, E), some strains contain four (3). The gene encoding this protein (*spa*) is different in length in various strains of the bacterium, between 1150 to 1500 base pairs (bp). Another reason for the* spa *gene length difference is its X region diversity; there is a repeated unit composed of a 24-nucleotide region on the X region of the* spa *gene, repeated 2-16 times. These factors can cause major polymorphisms, used as the base of *S. aureus* classification by a high differentiation power (4, 5). Due to the high precision and discriminatory power of this method as well as wide variety of the* spa *types, today, several online sites are designed to identify the *S. aureus* type, such as NCBI and* spa *Ridom type.

However, one of the problems in these methods is need for sequencing, which is expensive. In addition, in this typing method, only X region of the* spa *gene is considered, and other changes are forgotten, especially in the antibody binding site. Therefore, in this study, we sought to replace the X sequencing using PCR-RFLP (polymerase chain reaction restriction fragment-length polymorphism) method for the* spa *typing (6).

PCR-RFLP of* spa *for tracking and typing methicillin-resistant *S. aureus* (MRSA) was proposed in 2005 by Mitani (7). Different Enzymes, especially HaeII, have been used for this purpose; but in this study, we digested the* spa *gene product with Bsp143I enzyme. The OLIGO software version 5 was applied to detect the restriction sites of this enzyme on the* spa *gene. The restriction sites are outside of the X region and digest the GATC sequence. In wild type isolates, there are at least three restriction sites and we can find four bands in electrophoresis, according to the standard *S. aureus* strain 8325.

## 2. Objectives

This study was designed for *S. aureus* typing based on PCR-RFLP of the* spa *encoding gene, using the Bsp143I enzyme.

## 3. Patients and Methods

### 3.1. S. aureus Isolation

This study was carried out on 128 isolated *S. aureus* samples collected from nose of 53 (41.4%) healthcare workers as healthy carriers as well as 75 (58.6%) from patients referred either to the teaching hospital or private medical diagnostic laboratory in Gorgan, north of Iran. Clinical samples were obtained from 24, 16, 17 and 18 cases of urine, wound, blood and other specimens (including synovial fluid, sputum and throat culture), respectively. Purification and determination of *S. aureus* were performed by biochemical methods, and its identification based on amplification of the glutamate synthetase gene was confirmed which was performed by PCR. Specific primers of mecA were applied to assess the MRSA strains; it was found that 45 (35.2%) and 83 (64.8%) of *S. aureus* isolates were MRSA and methicillin-sensitive *S. aureus* (MSSA), respectively (8).

### 3.2. DNA Extraction and PCR of spa

DNA extraction and* spa *gene amplification were carried out according to our previous study (8) which can be explained briefly as follows: DNA was extracted using lysostaphin and phenol-chloroform, and specific primers (4) (mentioned below) were used for PCR with product lengths varying between 1150-1500 bp;

*spa*1) 5′-ATCTGGTGGCGTAACACCTG-3′
*spa*2) 5′-CGCTGCACCTAACGCTAATG-3′

### 3.3. PCR RFLP

The Bsp143I enzyme (Fermentas, Germany) was used to digest the final PCR product of the* spa *gene. The procedures were according to the instructions of Fermentas Co.: 10 μL (~ 0.1-0.5 μg of DNA) of PCR reaction mixture was added to 18 μL of nuclease-free water, 2 μL of 10X buffer x Bsp143I buffer, and 2 μL of Bsp143I. The mixture was mixed gently and spin down for a few seconds and incubated at 37°C for four hours. Afterwards, Bsp143I was inactivated by incubation at 65°C for 20 minutes. The OLIGO software version 5 was also applied to detect the restriction sites on the gene. The restriction sites of this enzyme are outside the X region and it recognizes the “GATC” sequence. The final products after Bsp143I digestion were analyzed by electrophoresis on 1.5% gel. Finally, they were compared with *S. aureus* strain 8325-4 on NCBI.

### 3.4. Statistical Analysis

Statistical comparison of the results was performed by χ^2^ test and P < 0.05 was considered significant.

## 4. Results

**Figure 1.**
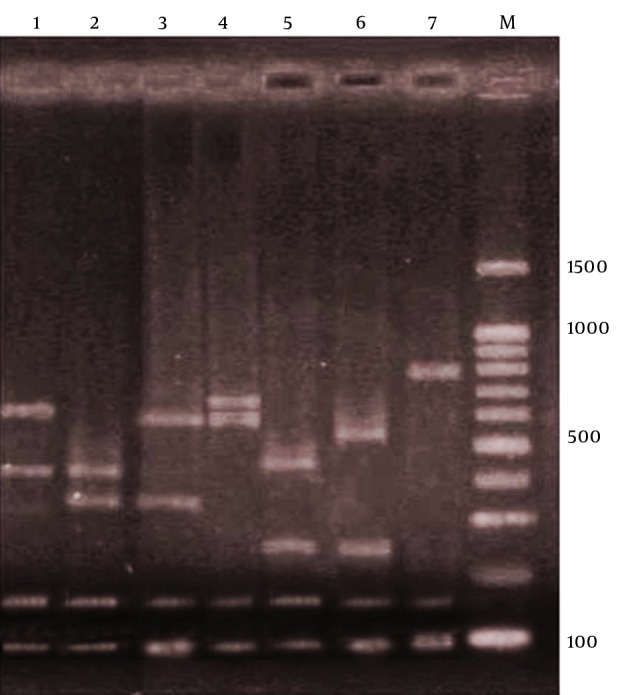
PCR Products Patterns of *S. aureus spa *Gene, After Digestion With Bsp143I Restriction Enzyme Lines 1 to 7 indicate the first to seventh types of *spa *and M is the 100-bp DNA ladder.

After PCR amplification, digestion of the* spa *gene by Bsp143I revealed seven types. Electrophoresis of the* spa*gene digestion showed four bands in six of the types, but in the seventh type, three bands were produced. Bands of 97 and 150 bp were present in all the seven types, but the sizes of other bands varied (Figure 1).

First and second* spa *gene types with 50 (39.06%) and 37 (28.9%) cases respectively, were the most common types, but only one *S. aureus* isolate belonged to the fifth to seventh* spa *types. The frequencies of the first to fourth* spa *types were as follows; type 1: 39.06%, 2: 28.9%, 3: 16.4%, and 4: 13.3%. Table 1 shows the distribution of seven* spa *types in MRSA and MSSA *S. aureus *samples, showing a statistically significant difference (P < 0.001). While type 1 (45.8%) was the most common among the MSSA isolates, type 2 (53.3%) was the most prevalent among MRSA isolates. On the other hand,* spa *type 3 was significantly more prevalent in MRSA than MSSA (Table 1). Table 2 shows the distribution of the seven* spa *types among *S. aureus* samples isolated from patients and healthy carriers.* spa *types 1 and 2 and *spa *types 1, 3 were predominant in *S. aureus* samples isolated from patients and healthy carrier, respectively. Difference in distribution of the* spa *types in the two groups was statistically significant (P = 0.003). Table 3 shows the frequency of all* spa *types in *S. aureus* samples isolated from the patients. While types 1, 2 and 4 were found in all the clinical samples, type 3 was only seen in the isolates from skin and throat culture.

**Table 1.** Distribution of the* spa *Types Among *S. aureus* Samples Isolated From Patients as Well as Carrier Strains, According to PCR-RFLP Method Using Bsp143I Enzyme ^a^

Type	1	2	3	4	5	6	7	Total
**Carrier ** ^**b**^	17 (32.1)	11 (20.8)	17 (32.1)	7 (13.2)	0	0	1 (1.9)	53 (100.0)
**Patient**	33 (44.0)	26 (34.7)	4 (5.3)	10 (13.3)	1 (1.3)	1 (1.3)	0	75 (100.0)
**Total**	50 (39)	37 (28.9)	21 (16.4)	17 (13.3)	1 (0.8)	1 (0.8)	1 (0.8)	128 (100)

^a^ Data are presented in No. (%).

^b^ P value of the* spa *types between carriers and patients was = 0.003.

**Table 2.** Distribution of the* spa *Types Among *S. aureus* Samples Isolated From MRSA and MSSA Strains, According to PCR-RFLP Method Using Bsp143I Enzyme ^a,b^

Type	1	2	3	4	5	6	7	Total
**MSSA ** ^**c**^	38 (45.8)	13 (15.7)	20 (24.1)	9 (10.8)	1 (1.2)	1 (1.2)	1 (1.2)	83 (100.0)
**MRSA**	12 (26.7)	24 (53.3)	1 (2.2)	8 (17.8)	0	0	0	45 (100.0)
**Total**	50 (39)	37 (28.9)	21 (16.4)	17 (13.3)	1 (0.8)	1 (0.8)	1 (0.8)	128 (100)

^a^ Abbreviations: MRSA, methicillin-resistant *S. aureus*; MSSA, methicillin-sensitive *S. aureus*.

^b^ Data are presented in No. (%).

^c^ P value of the* spa *types between MRSA and MSSA was < 0.001.

**Table 3.** Frequencies of the Types Isolated From the Patients ^a^

Type	Urine	Wound	Blood	Others ^b^	Total
**1**	12 (36.3)	4 (12.1)	6 (18.1)	11 (33.3)	33 (100)
**2**	6 (23.1)	7 (26.9)	9 (34.6)	4 (15.3)	26 (100)
**3**	0	3 (75)	0	1 (25)	4 (100)
**4**	5 (50)	1 (10)	2 (20)	2 (20)	10 (100)
**5**	0	1 (100)	0	0	1 (100)
**6**	1 (100)	0	0	0	1 (100)
**7**	0	0	0	0	0
**Total**	24 (32)	16 (12.5)	17 (12.9)	18 (13.6)	75 (100)

^a^ Data are presented in No. (%).

^b^* S. aureus* isolated from other samples such as: synovial fluid, CSF, sputum, throat culture, and stool culture.

## 5. Discussion

Typing of pathogenic bacteria is an important process in epidemiological studies and control of nosocomial infections.* spa *typing and PCR-RFLP in addition to pulsed field gel electrophoresis (PFGE) are among the efficient methods for differentiating *S. aureus*, particularly MRSA strains (9). In this study, *S. aureus* isolates were classified by PCR-RFLP of the* spa *gene with Bsp143I enzyme, which classified in seven types.

The* spa *gene lengths in the isolated cases varied from 1150 to 1500 bp, which were slightly longer than the data reported from a study in India (1150-1420 bp). The maximum repetition of the X-region in our samples was 12 times, but the same item in a study from India was 13 (4). This discrepancy is probably due to differences in the IgG binding site of the* spa *gene, which is outside of the X region. Bsp143I is an endonuclease enzyme that digests the GATC sequence in DNA, which is used in several studies (10, 11). However, this is the first time this enzyme is applied for digestion of the* spa *gene for *S. aureus* typing. Using this method, we were able to divide the *S. aureus* isolates to seven types. In a similar study in India on 149 isolates of *S. aureus*, PCR product of the* spa *gene was digested with HaeII enzyme and five types were identified. In other studies using HaeII enzyme in Germany and Iran, 14 and seven types were diagnosed, respectively (4, 12).

These findings imply that efficacy of the Bsp143I enzyme in digesting the *S. aureus spa *gene is similar to HaeII enzyme. In accordance with a previous research, we found that variation of the *spa *types among MSSA is more than MRSA isolates, insomuch all the seven* spa *types were present in MSSA isolates, but only four (types1-4) were seen in MRSA. Fenner et al. diagnosed 65 and 42* spa *types among 101 MSSA and 200 MRSA isolates, respectively (13). Strommenger et al. demonstrated that the types variations among 283 MSSA isolates (128 types) was more than the variations among 1176 MRSA isolates (121 types) (14). Methicillin resistance is a relatively new phenomenon caused by acquiring the mecA gene by *S. aureus*; thus, many strains of *S. aureus* may not still have enough time to acquire the resistance genes. On the other hand, believing that only some *S. aureus* strains have the ability to obtain the mecA gene, just certain types of *S. aureus* can be MRSA and diversity of the* spa *types is less than MSSA (8).

Variety of the *S. aureus* strains *spa *types isolated in this study among patients and healthy carriers was statistically significant. Although distribution of the type 1* spa *gene was similar in both patients and healthy carriers, the frequency of type 3 among carriers was significantly more.

This probably means fewer virulence of type 3 *spa*. Although it easily colonized in the noses of health care workers, it was not important in the pathogenesis. Further studies are recommended to identify its frequency and possible reasons for its decreased pathogenicity.

Our results showed that the Bsp143I enzyme is a useful tool for digestion of the* spa *gene and typing of *S. aureus*. The *spa *types variations among MSSA were more than MRSA isolates.
